# Gene Expression Profiling of Lymphoblasts from Autistic and Nonaffected Sib Pairs: Altered Pathways in Neuronal Development and Steroid Biosynthesis

**DOI:** 10.1371/journal.pone.0005775

**Published:** 2009-06-03

**Authors:** Valerie W. Hu, AnhThu Nguyen, Kyung Soon Kim, Mara E. Steinberg, Tewarit Sarachana, Michele A. Scully, Steven J. Soldin, Truong Luu, Norman H. Lee

**Affiliations:** 1 Department of Biochemistry and Molecular Biology, The George Washington University Medical Center, Washington, D. C., United States of America; 2 Departments of Medicine, Pharmacology, and Oncology, Georgetown University Medical Center, Washington, D. C., United States of America; 3 Department of Pharmacology and Physiology, The George Washington University Medical Center, Washington, D. C., United States of America; National Institutes of Health, United States of America

## Abstract

Despite the identification of numerous autism susceptibility genes, the pathobiology of autism remains unknown. The present “case-control” study takes a global approach to understanding the molecular basis of autism spectrum disorders based upon large-scale gene expression profiling. DNA microarray analyses were conducted on lymphoblastoid cell lines from over 20 sib pairs in which one sibling had a diagnosis of autism and the other was not affected in order to identify biochemical and signaling pathways which are differentially regulated in cells from autistic and nonautistic siblings. Bioinformatics and gene ontological analyses of the data implicate genes which are involved in nervous system development, inflammation, and cytoskeletal organization, in addition to genes which may be relevant to gastrointestinal or other physiological symptoms often associated with autism. Moreover, the data further suggests that these processes may be modulated by cholesterol/steroid metabolism, especially at the level of androgenic hormones. Elevation of male hormones, in turn, has been suggested as a possible factor influencing susceptibility to autism, which affects ∼4 times as many males as females. Preliminary metabolic profiling of steroid hormones in lymphoblastoid cell lines from several pairs of siblings reveals higher levels of testosterone in the autistic sibling, which is consistent with the increased expression of two genes involved in the steroidogenesis pathway. Global gene expression profiling of cultured cells from ASD probands thus serves as a window to underlying metabolic and signaling deficits that may be relevant to the pathobiology of autism.

## Introduction

Autism spectrum disorders (ASD, or the “autisms”) are a group of pervasive neurodevelopmental disorders that are characterized by delayed or abnormal development and use of language, poor reciprocal social interactions, restricted interests and repetitive behaviors [1]. Although the etiology of ASD is not known, the core symptomatology has focused much of autism research on the brain [2]–[4]. At the same time, there is increasing evidence for the involvement of other organ systems, especially the immune and gastrointestinal systems, in individuals affected by ASD [5]–[10]. The interactions of these systems with the nervous system are likely mediated by biologic factors, such as hormones and cytokines that, in turn, may be induced by environmental stressors [11]–[17]. Of particular interest, Jyonouchi et al. demonstrated a relationship between the immune and gastrointestinal symptoms in children with ASD by showing activation of the innate immune response (rise in TNF-α levels in response to lipopolysaccharide) in peripheral blood mononuclear cells only in ASD children with positive gastrointestinal symptoms [9]. The connection between gastrointestinal symptoms and ASD was strengthened by genetic studies which showed an association between a single nucleotide polymorphism (SNP) in the promoter of the MET gene (which is an oncogene involved in cerebellar growth, immune function, and gastrointestinal repair) and autism [18]. The observed reduction of the MET protein in autistic brain samples relative to matched control brain samples further supported the involvement of MET in ASD [19]. Recently, neural inflammation and oxidative stress have been proposed as possible contributing factors to the etiology of autism [20]–[23]. In particular, Vargas et al. [12] demonstrated neuroglial activation and presence of inflammatory cytokines in the brain of autistic patients while Chauhan et al. [24] showed evidence for lipid peroxidation and reduction of antioxidant proteins in autism. In exploring the molecular bases of oxidative stress in ASD, James et al. studied the metabolites involved in the methionine transmethylation and transsulfuration pathways and found significant reduction in the levels of methionine, S-adenosylmethionine, cysteine, and free reduced glutathione which are reflective of oxidative stress, and further demonstrated genetic variants in several of the genes within these pathways in individuals with ASD [22]. Taking an animal model approach to exploring possible metabolic contributions to the pathogenesis of autistic-like behavior, MacFabe et al. investigated the effects of propionic acid (PPA), a short chain fatty acid and an important intermediate of cellular metabolism, on behavior, electrographic activity, neuropathology, and oxidative stress in rats exposed to intraventricular PPA [23]. Interestingly, PPA is also a fermentation by-product of a subpopulation of opportunistic enteric bacteria (eg., clostridia, propionibacteria), which have been cited as a putative risk factor for ASD [25]. The results of these studies [23], [26] show that PPA not only induced behaviors characteristic of autism (eg., repetitive dystonic behaviors, retropulsion, seizures, as well as social avoidance), but also replicated the pathological findings associated with ASD, such as neuroinflammation, reactive astrogliosis, and activated microglia, in addition to lipid and protein oxidation and reduction of total glutathione in brain homogenates which are collectively indicators of oxidative stress. These limited examples demonstrate that, although the most noticeable impairments in autism are those affecting higher order neurological functions, there is increasing evidence, recently reviewed by Zecavati and Spence [27], that many “neurometabolic disorders” are also associated with the phenotype of ASD. Thus, the heterogeneity of behavioral, functional, physiological, and metabolic manifestations of ASD combined with the possible contribution of multiple organ systems to the clinical complexity of autism suggests that a more global, systems approach may be needed to elucidate the molecular underpinnings of ASD in defined phenotypic subgroups of patients.

### Genetic approaches to identify genes associated with autism

Although ASD is the most heritable of all the psychiatric disorders based upon twin and family studies [28]–[31], no single gene or consensus gene combination has been shown to be causally linked to *idiopathic* autism. At present, genetic susceptibility loci have been identified on virtually every chromosome by a combination of whole genome scans, cytogenetics, and genetic linkage/association analyses [32]–[35]. Although there is great diversity in the genes identified as potential candidates, synapse formation/function and axon guidance are emerging as principal functional themes in ASD from recent studies. The respective genes include glutamate receptors, GABA receptors, neuroligins, neurexin 1, and SHANK3 which are involved in synapse formation and function [36]–[41], and RELN, ROBO1/2, SLIT2, ITGB1, PAK, and MET which are involved in axon guidance [18], [42]–[44]. Additional genes include those involved in development, neuronal differentiation, and survival, such as WNT2, HOXA1, and BCL2 [42], [45], [46]. Part of the difficulty in conclusively identifying and confirming genes for autism is thought to arise from the heterogeneity of phenotypes of ASD combined with the relatively small sample sizes in the various studies. A recently completed large-scale SNP association analysis involving 1,168 multiplex families revealed a single region on chromosome 11p12-p13 that exceeded the threshold for suggestive linkage when data from all of the families was combined [47]. It is interesting that none of the previously identified candidate genes were located in this region. On the other hand, this study and another demonstrated for the first time that copy number variants (some of which are *de novo*) are 10 times more frequent in the autistic population than in the general population [48], suggesting the contribution of epigenetic or environmental factors to ASD.

### Genomic approaches to investigation of ASD

Genomic methods involve simultaneous, large-scale expression analysis of thousands of genes on a cDNA (or oligonucleotide) microarray slide [49], [50]. The first DNA microarray study of autism identified ∼30 genes as differentially expressed in the cerebellum from autopsy tissue of autistic and normal subjects, and focused on the abnormal expression of the glutamate receptor as a potential pathogenic mechanism [51]. Several recent applications of global gene expression analysis to autism have evaluated gene expression in lymphoblastoid cell lines (LCL) and in whole blood with the goal of identifying differentially expressed genes in a peripherally-derived tissue which may serve as diagnostic biomarkers for ASD, or surrogate markers for dysregulated metabolic and signaling pathways in peripheral and central tissues, which may provide clues to the pathophysiology of the disorder [43], [44], [52]–[54]. Our previous study on LCL from monozygotic twins discordant in severity of ASD revealed differentially expressed genes that function in nervous system development as well as genes that mapped *in silico* to autism susceptibility regions on chromosomes that were previously identified by numerous genetic analyses [43]. A recent study by Geschwind and colleagues, which compared the gene expression profiles of LCL from individuals with known genetic causes of autism (Fragile X and 15q11-q13 duplication), identified 68 commonly dysregulated genes, two of which (JAKMIP1 and GPR155) were also confirmed as differentially expressed in LCL from male sib pairs discordant for idiopathic autism [53]. These studies collectively demonstrate the power of applying a genomic approach based on global gene expression to identify genes that may be involved in common pathways giving rise to ASD.

In this study, we postulate that differentially expressed genes in autistic vs. normal nonautistic siblings are at least in part responsible for the autism phenotype, as we had earlier shown for monozygotic twins discordant in diagnosis of autism [43]. We therefore analyzed gene expression profiles of LCL derived from 21 sib pairs where one of the siblings is autistic and the other is not. To reduce the phenotypic heterogeneity among the samples, we selected cell lines from individuals who presented with severe language impairment as reflected by scores on the Autism Diagnostic Interview-Revised (ADIR) questionnaire, as described in Materials and Methods. Results from gene expression analysis of LCL from these individuals revealed alterations in genes involved in cholesterol metabolism and steroid hormone biosynthesis, as well as genes involved in neuronal processes and development. A steroid profile of cell extracts using HPLC-tandem mass spectrometry methods further confirmed elevations in testosterone levels from a subset of the autistic and their respective unaffected siblings.

## Results

### Differentially expressed genes between autistic probands and sibling controls implicate steroid biosynthetic pathways

**Figure 1:**
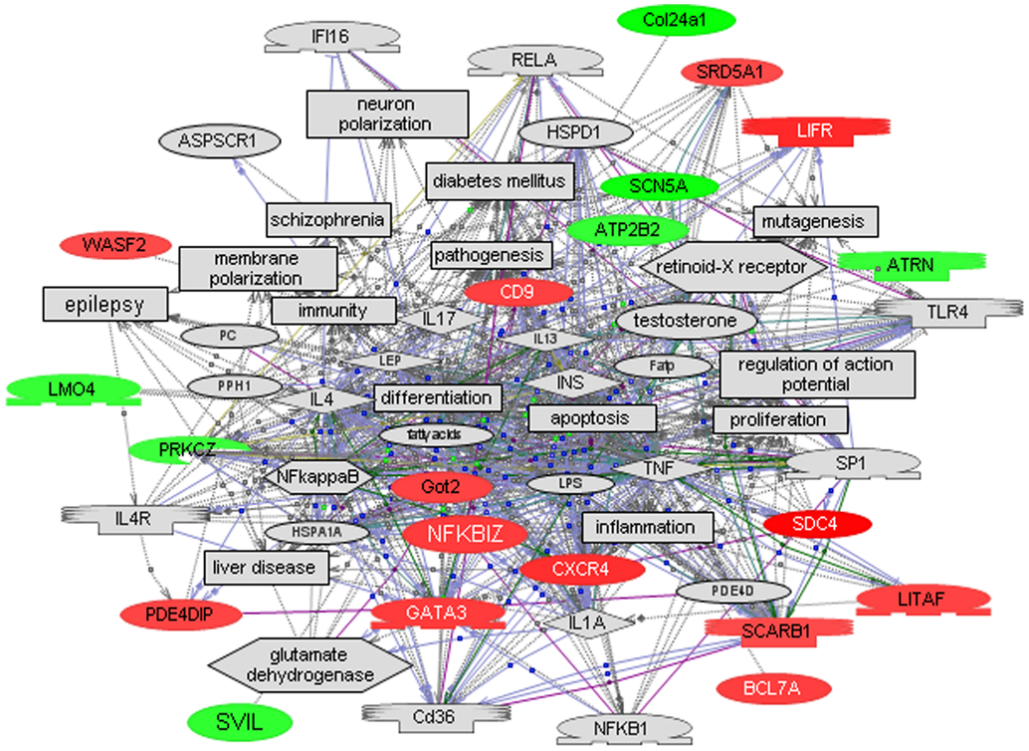
A relational gene network constructed using Pathway Studio 5 from the dataset of significant genes identified by SAM analysis with 70% data filtering (see Table 1). Red denotes genes with increased expression in the autistic sibling while green indicates decreased expression. Note that inflammation, epilepsy, liver disease, diabetes, and schizophrenia are among the pathological processes associated with this gene network while apoptosis, differentiation, and regulation of action potential are among the cellular processes that are influenced by this set of genes.

The log2 ratios of relative gene expression from autistic and nonautistic siblings were analyzed by one-class SAM using 70% data filtering, which requires that at least 70% of the samples must have non-zero expression ratios in order for a given gene to be included in the statistical analysis. Significant differentially expressed genes with a log2 ratio≥±∼0.3 are shown in **Table 1**. This expression cutoff was selected on the basis of our ability to confirm genes exhibiting this level of differential expression by qRT-PCR analysis. **Figure 1** shows the major multigene interaction network constructed by Pathway Studio 5 software which comprises genes that were differentially expressed between normal and autistic siblings. Interestingly, this network includes cellular (apoptosis, differentiation) [43] and disease processes such as inflammation [12], [13] and epilepsy [55], [56] that are often associated with ASD [57]. **Table 2** lists the top 3 (out of 69) high level functions that were identified by Ingenuity Pathways Analysis as being significantly overrepresented by differentially expressed genes in this dataset. SCARB1 and SRD5A1, which are represented in the top 2 functions (endocrine system development and function and small molecule biochemistry), implicate involvement of the steroid hormone biosynthetic pathway. This is further supported by Pathway Studio 5 analysis which shows that steroid hormones as well as neurotransmitters are an integral part of a network of common regulators and targets of this set of differentially expressed genes (**Table 3**). Among the disorders that are identified as common targets are inflammation, epilepsy, diabetes mellitus, digestive disorders, and hyperandrogenemia, all of which have been associated with ASD. Of particular relevance to these pathway-implicated disorders are the findings of elevated inflammatory cytokines in autopsy brain tissue from autistic patients relative to controls as well as increases in IFN-gamma and IL-1RA in whole blood of autistic children [12], [13]. Of the genes in the relational network shown in Fig. 1, SCN5A, a cardiac Na+-gated sodium channel also expressed in the limbic brain, may be associated with seizures [58]. Epilepsy and/or epileptiform EEG abnormalities are comorbid disorders that variably affect from 5 to 46% of the autistic population [55], and a population-based study further suggests that the prevalence of ASD is higher in children who experience seizures in the first year of life [56]. Interestingly, kindled limbic seizures as well as autistic-like motor and social behaviors have been induced in a rat model by intraventricular administration of PPA, a short-chain fatty acid [23], [26], and this animal model was further demonstrated to exhibit evidence of neuroinflammation and oxidative stress in specific brain regions [59]. It is noteworthy that PPA is also the metabolic end product of enteric bacteria [60], and that diabetes and digestive disorders, which are among the targets implicated by our differentially expressed genes, have also been associated with the autistic population [5]–[7], [61]–[63]. The suggested association of hyperandrogenemia is especially interesting as females with congenital adrenal hyperplasia which results in elevated levels of testosterone were shown to exhibit higher autistic-like social behavior [64], and conversely, women affected by ASD as well as their mothers exhibited more masculine physical and behavioral traits, suggestive of higher androgen levels [14]. These findings thus support the argument that autism may in part be considered a systemic encephalopathic condition involving immune, digestive and metabolic/endocrine dysfunction, which may be exacerbated by environmental triggers in genetically sensitive subpopulations. Gene ontological analysis using Database for Annotation, Visualization and Integrated Discovery (DAVID) software [65] further demonstrated statistically significant enrichment of differentially expressed genes involved in nervous system development as well as organization and biogenesis of the actin cytoskeleton (**Table 4**).

**Table 1:** Differentially expressed genes between autistic and control siblings (FDR = 13.5%).

Genbank #	Gene Symbol	log2(ratio)*	SEM
AI421603	ATP2B2	−0.34	−0.09
N80619	ATRN	−0.32	−0.08
H90147	BCL7A	0.36	0.08
AA973009	C16ORF44	0.32	0.07
AI291693	C21ORF34	−0.48	−0.11
AA932364	CCDC102B	0.31	0.07
AA412053	CD9	0.35	0.08
N51674	COL24A1	−0.65	−0.14
AA458486	COMMD4	0.33	0.08
T62491	CXCR4	0.47	0.1
AA410291	FGD6	0.36	0.08
AA025380	GATA3	0.38	0.08
AA487739	GOT2	0.34	0.09
AA884151	GPR175	0.34	0.09
AI283902	HIST1H1A	0.34	0.09
AA007370	HKR1	0.37	0.9
AA984306	HMBOX1	0.3	0.07
AA609471	IER5L	0.54	0.13
AA115054	KCTD12	−0.32	−0.07
H10192	LIFR	0.51	0.11
AA625666	LITAF	0.34	0.07
AA644559	LMO4	−0.33	−0.07
R83847	LOC388335	0.37	0.83
AA256157	MIRH1	0.29	0.06
H69786	NFKBIZ	0.38	0.08
H15535	PDE4DIP	0.33	0.07
AI221690	PRKCZ	−0.3	−0.07
AI198213	RNU12P	−0.55	−0.12
AA443899	SCARB1	0.29	0.06
AA044664	SCN5A	−0.61	−0.16
AA148736	SDC4	0.87	0.21
R36874	SRD5A1	0.29	0.06
AI291307	SVIL	−0.36	−0.08
N73575	TRIM25	0.41	0.09
AA429572	WASF2	0.29	0.07
R28287	unknown	0.41	0.09
AI061421	unknown	0.35	0.08
R32996	unknown	0.33	0.08
H20826	unknown	0.31	0.07
AI217709	unknown	0.3	0.07
R12679	unknown	0.29	0.07
AA424531	unknown	−0.31	−0.07
AI141767	unknown	−0.32	−0.08
AI122714	unknown	−0.5	−0.13
AA939238	unknown	−0.65	−0.14

**Table 2:** Biological functions identified by Ingenuity Pathway Analysis of significant differentially expressed genes (log2 ratio>±0.3) identified by SAM analysis (FDR = 13.5%).

Category	Function Annotation	p-value*	Molecules
Endocrine System Development and Function	biosynthesis of androgen/steroidogenesis	4.89E-05	SCARB1, SRD5A1
	proliferation of pancreatic duct cells	3.69E-03	CXCR4
	quantity of 4-androstene-3,17-dione	1.29E-02	SRD5A1
Small Molecule Biochemistry	endocytosis of cholesterol	1.85E-03	SCARB1
	breakdown of progesterone	1.85E-03	SRD5A1
	biosynthesis of norepinephrine	5.53E-03	GATA3
	synthesis of ganglioside GM3	7.37E-03	CD9
	uptake of taurocholic acid	1.83E-02	PRKCZ
Nervous System Development and Function	morphology of neurons	5.49E-04	CD9, GATA3
	morphology of Purkinje cells	1.85E-03	ATP2B2
	morphology of serotonergic neurons	1.85E-03	GATA3
	fusion of vagus cranial nerve ganglion	1.85E-03	LMO4
	polarization of astrocytes	1.85E-03	PRKCZ
	development of cerebellum	1.98E-03	ATP2B2, CXCR4
	branching of sympathetic neuron	3.69E-03	LIFR
	differentiation/quantity of central nervous system cells	5.43E-03	ATP2B2, LIFR
	morphology of central nervous system	5.53E-03	ATRN
	development of Purkinje cells	1.10E-02	CXCR4
	migration of motor neurons	1.83E-02	GATA3
	biogenesis of synapse	2.74E-02	ATP2B2
	guidance of motor axons	2.74E-02	CXCR4

* Significance calculated for each function is an indicator of the likelihood of that function being associated with the dataset by random chance. The range of p-values was calculated using the right-tailed Fisher's Exact Test, which compares the number of user-specified genes to the total number of occurrences of these genes in the respective functional/pathway annotations stored in the Ingenuity Pathways Knowledge Base.

**Table 3:** Common regulators and targets of differentially expressed genes (from Table 1) identified by Pathway Studio 5 analysis.

Regulators	Targets
**Small molecule**	**Small molecule**
androgen	androgen
Ca2+	androstenedione
cAMP	Ca2+
cholesterol	cAMP
dexamethasone	ceramide
estradiol	cholesterol
estrogen	cortisol
fatty acids	estradiol
glucocorticoid	estrogen
glucose	fatty acids
norepinephrine	glutamate
phospholipids	lipid
progesterone	melanin
RA	NO
siRNA	progesterone
steroids	testosterone
testosterone	tyrosine
**Disorders**	**Disorders**
diabetes mellitus	Crohn disease
fetal development	dementia
inflammation	diabetes mellitus
neural tube malformation	digestion
neuroblastoma	embryonic cell viability
	endocrine abnormality
	endocrine function
	epilepsy
	fetal development
	hyperandrogenemia
	hyperinsulinemia
	inflammation
	long-term potentiation
	muscular dystrophy
	neural tube malformation
	neuron dysfunction
	neuron toxicity
	peripheral nerve function

**Table 4:** Gene ontology analysis using DAVID of significant differentially expressed genes with (log2 ratio>±0.3) identified by SAM analysis (FDR = 13.5%).

Process	p-value*	Genes
GO:0008092∼cytoskeletal protein binding	3.46E-03	SVIL, PDE4DIP, WASF2, CXCR4, SDC4,
GO:0030036∼actin cytoskeleton organization and biogenesis	4.18E-03	SVIL, PDE4DIP, WASF2, FGD6,
GO:0030029∼actin filament-based process	5.06E-03	SVIL, PDE4DIP, WASF2, FGD6,
GO:0007010∼cytoskeleton organization and biogenesis	8.38E-03	PRKCZ, SVIL, PDE4DIP, WASF2, FGD6,
GO:0003779∼actin binding	9.61E-03	SVIL, PDE4DIP, WASF2, CXCR4,
GO:0006996∼organelle organization and biogenesis	3.47E-02	PRKCZ, SVIL, PDE4DIP, HIST1H1A, WASF2, FGD6,
GO:0016043∼cellular component organization and biogenesis	4.89E-02	PRKCZ, ATP2B2, SVIL, PDE4DIP, HIST1H1A, WASF2, CXCR4, CD9, FGD6,
GO:0032502∼developmental process	4.01E-04	PRKCZ, SRD5A1, SVIL, HKR1, CD9, SCARB1, FGD6, LITAF, GPR175, ATP2B2, LMO4, CXCR4, ATRN, GATA3,
GO:0000003∼reproduction	9.35E-03	SRD5A1, ATP2B2, HIST1H1A, CXCR4, CD9,
GO:0065007∼biological regulation	1.28E-02	PRKCZ, BCL7A, SVIL, HKR1, CD9, LIFR, HMBOX1, FGD6, SCN5A, LITAF, ATP2B2, LMO4, CXCR4, ATRN, GATA3,
GO:0009653∼anatomical structure morphogenesis	2.83E-02	ATP2B2, LMO4, CXCR4, CD9, FGD6, GATA3,
GO:0007399∼nervous system development	3.12E-02	ATP2B2, LMO4, CXCR4, CD9, GATA3,
GO:0048646∼anatomical structure formation	3.15E-02	ATP2B2, CXCR4, CD9,

* Significance by Fisher's Exact Test.

### Confirmation of differentially expressed genes related to steroid metabolism, inflammation, and nervous system development by qRT-PCR analysis

**Figure 2:**
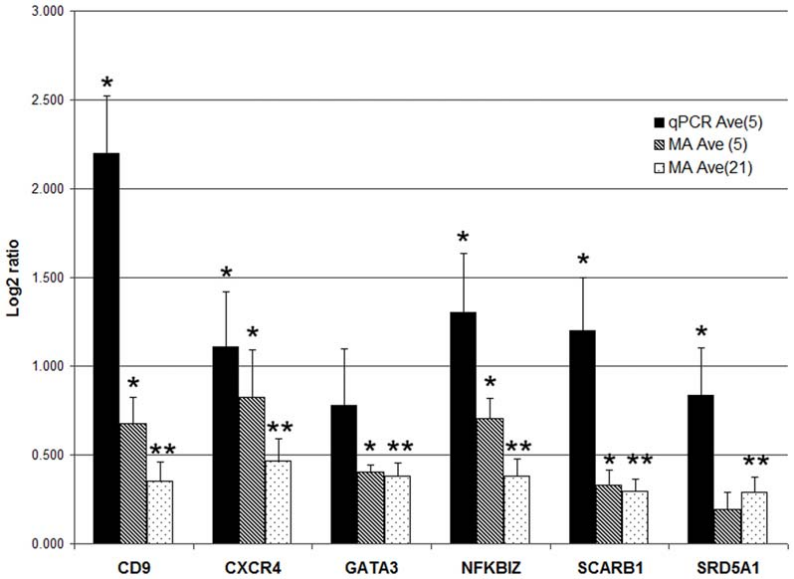
Confirmation of select differentially expressed genes by qRT-PCR analyses. Five representative samples were analyzed per group for each gene, with each sample run in triplicate. The graph shows the average log2 ratios obtained for each gene for the 5 samples analyzed by qRT-PCR, for the same 5 samples analyzed by DNA microarrays, and for all 21 samples analyzed by DNA microarrays. *p-value<0.05; **p-value<0.004; The p-values for the qRT-PCR analysis of GATA3 was 0.069, and for the microarray analysis of SRD5A1 based on only 5 samples was 0.156. However, the p-values for GATA3 and SRD5A1 based on the microarray analysis of all 21 paired samples were <0.00006 and <0.002, respectively.

**Figure 3:**
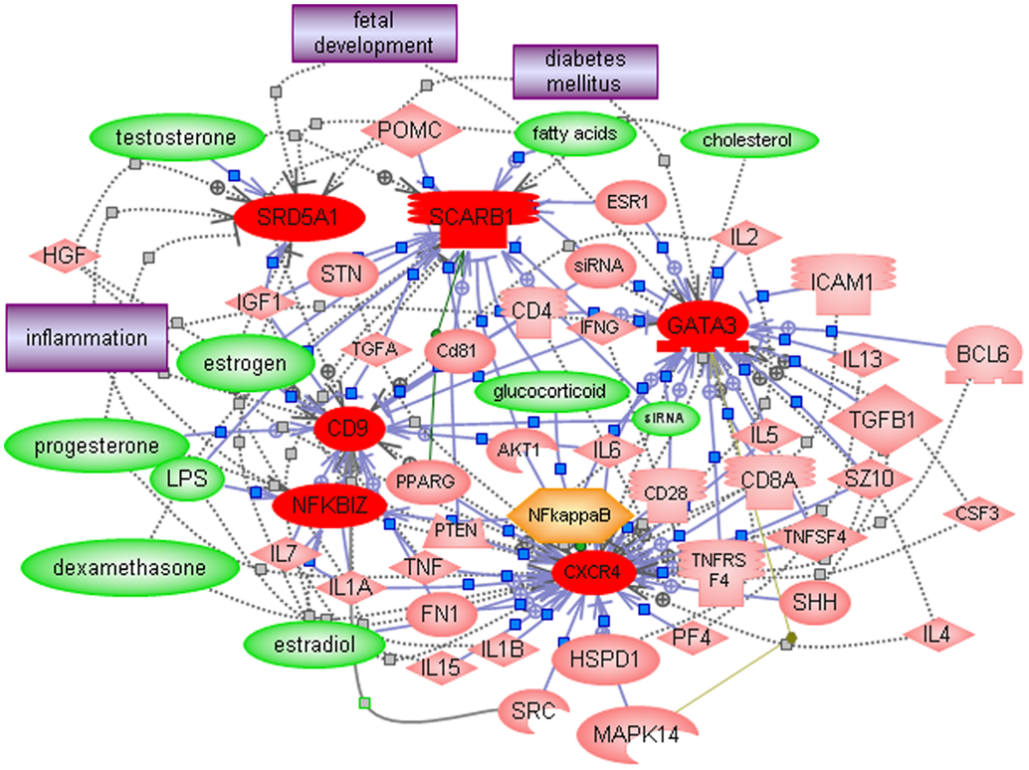
Common regulators (identified by Pathway Studio 5) associated with the dataset of significant differentially expressed qRT-PCR confirmed genes. Color coding of entities associated with gene network: Red – upregulated genes; pink – other genes which are part of the regulatory network constructed by the pathway analysis program; green - small molecules; orange - functional class; purple - disorders.

**Figure 4:**
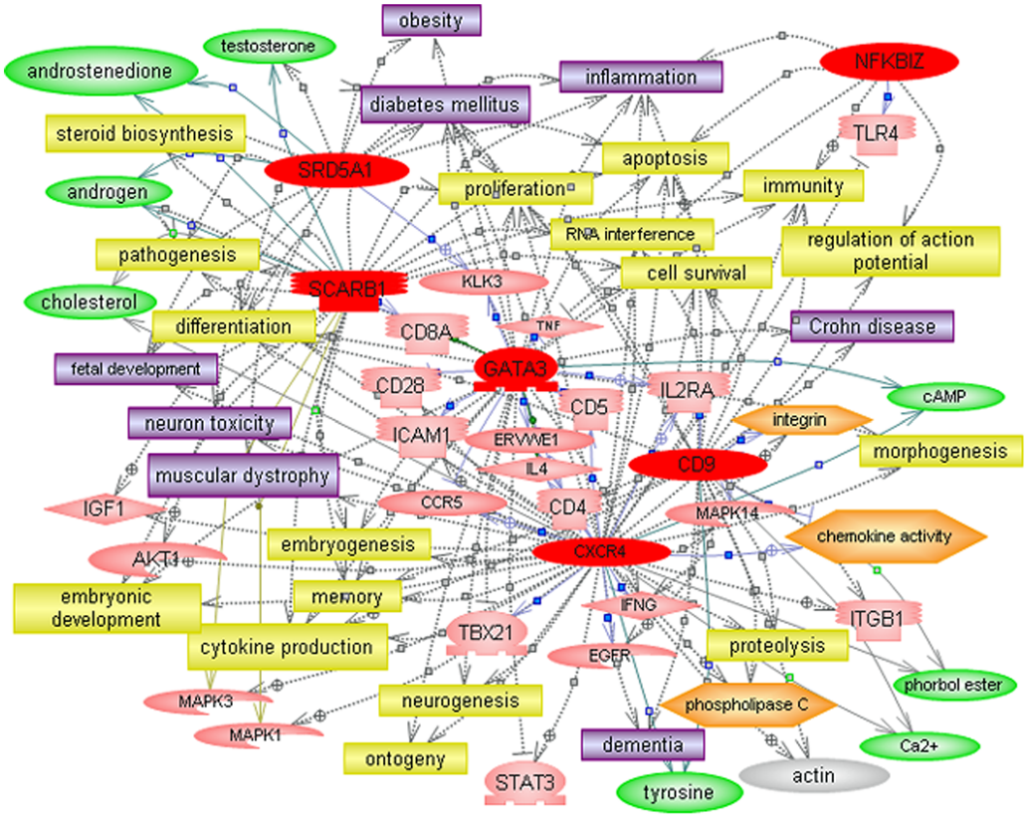
Common targets (identified by Pathway Studio 5) associated with the dataset of significant differentially expressed qRT-PCR confirmed genes. Colored entities are defined in legend to Fig. 3. Yellow entities describe cellular processes.

Quantitative RT-PCR (qRT-PCR) was used to confirm the differential expression of genes represented among the top biological functions (Table 2), including those involved in cholesterol/steroid hormone metabolism and several that are involved in development of the nervous system and inflammation (**Figure 2**). Pathway Studio 5 was then used to identify the common regulators as well as common targets of the six confirmed genes in order to examine their possible roles in the context of autism. It is noteworthy that cholesterol as well as several steroid hormones, including testosterone, progesterone, and estradiol are among the small molecule regulators of this network of genes (**Figure 3**), suggesting the possibility of feedback regulation between these metabolites and genes involved in their production. Indeed, cholesterol and androgenic hormones (testosterone and androstenedione) are among the common molecular targets of these 6 genes, which are also implicated in a number of dysfunctional processes (embryonic development, neurogenesis, apoptosis, cytokine production) and disorders (inflammation, digestive disorder, muscle disorder) associated with ASD (**Figure 4**). Aside from the novel candidate genes identified in this study, the networks in **Figs. 3** **and** **4** also include 2 other genes, ITGB1 and PTEN, which have been identified as candidate ASD genes in other studies [44], [66]–[68]. Of particular significance is that PTEN has been demonstrated to be downregulated in a mouse model of Purkinje cell degradation, a hallmark of autism neuropathology [69]. In addition, a mouse model involving limited deletion of PTEN in the cerebral cortex and hippocampus resulted in mice with macrocephaly, abnormal social interactions, and increased sensitivity to sensory stimuli, characteristics that are often associated with ASD [70].

### Steroid profiling reveals elevated testosterone levels in LCL extracts from autistic siblings

**Figure 5:**
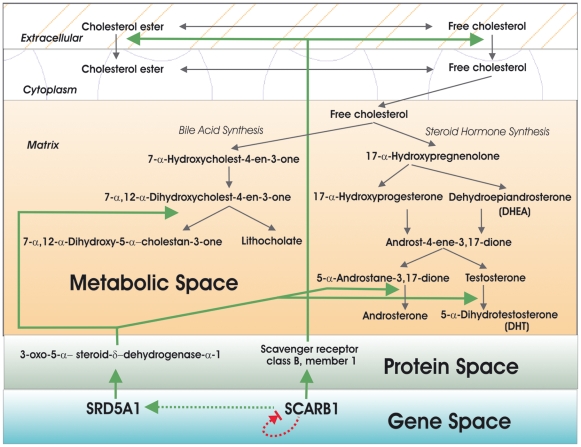
A bionetwork that shows the relationships and interactions between SCARB1 and SRD5A1 at the gene, protein, and metabolite levels. Briefly, SCARB1 is responsible for the uptake of cholesterol into cells while SRD5A1 converts testosterone to 5-α-dihydrotestosterone (DHT), a more potent form of the male hormone. We propose that increases in the expression of these genes may lead to an overall increase in the production of androgens. It is also of interest that bile acid synthesis is linked to this same pathway, thereby suggesting that altered expression of these genes in ASD may lead to disturbances of bile acid synthesis in some tissues as well.

Based upon the qRT-PCR-confirmed differential expression of SCARB1 and SRD5A1 which are involved in cholesterol metabolism and steroid hormone biosynthesis, we constructed a multilevel biomolecular network representing the possible interactions and functions of the genes, gene products, and downstream metabolites (**Figure 5**). From this bionetwork, we postulated that elevations in these genes may lead to an increase in androgenic hormone biosynthesis and conducted pilot steroid profiling analyses on LCL extracts from 3 randomly selected sibling pairs to test this hypothesis. Indeed, **Table 5** shows that testosterone was elevated in the extracts from all 3 autistic siblings relative to their respective non-autistic siblings. This data is consistent with other reports that androgenic hormones are elevated in serum from autistic individuals relative to that from normal age- and gender-matched controls [71] and provides further support for the role of elevated male hormones as a risk factor for autism [14], [17], [64], [72].

**Table 5:** Concentration of testosterone in LCL extracts from 3 pairs of autistic-nonautistic siblings as determined by HPLC-MS/MS analyses.

Sample	Age	Status	Testosterone (ng/dL)	Ratio (autistic/normal)
HI0366	18	autistic	241	1.14
HI0365	20	normal	212	
HI0355	12	autistic	218	≥218
HI0354	14	normal	<1	
HI2769	10	autistic	251	1.22
HI2772	13	normal	206	

* Below level of detection.

## Discussion

It is becoming increasingly clear that although the neurological symptoms of ASD are the most striking among the behavioral and functional manifestations of affected individuals, there are many associated peripheral physiological symptoms that have often gone unnoticed/ignored and clinically unaddressed. These include gastrointestinal disorders experienced by many on the spectrum (estimated at 50%) as well as immune disorders which have long been described in the literature on ASD [5]–[10]. The large-scale global gene expression profiling that we have undertaken on LCL derived from peripheral blood lymphocytes of ASD probands and their respective siblings may therefore serve as a window to the underlying biochemical and signaling deficits that may be relevant to understanding the broader symptomatology of autism.

Overall, our study of autistic-nonautistic sib pairs in which the autistic sibling has been subtyped according to severity of language impairment on the basis of cluster analysis of scores from the ADIR diagnostic interview (Hu and Steinberg, Autism Research, 2009, in press), reveals altered expression of genes that participate in cholesterol metabolism and androgen biosynthesis. It is noteworthy that deficiency of 7-dehydrocholesterol reductase (DHCR7), the terminal enzyme in cholesterol biosynthesis, is the genetic cause of Smith-Lemli-Opitz syndrome (SLOS) which is an autosomal recessive disorder characterized by pre- and post-natal growth retardation, distinct facial anomalies, microcephaly, and mental retardation [73], [74]. Significantly, over 50% of individuals with SLOS also meet the diagnostic criteria for autism [75], [76]. Although the mechanism through which DHCR7 deficiency causes any of these characteristics/phenotypes is unknown, a gene expression study of a DHCR7 knockout mouse model reveals altered expression of numerous genes affecting not only cholesterol biosynthesis, but also neurodevelopment and functions such as Wnt signaling, axon guidance, neuronal cytoskeletal assembly, and neurodegeneration [77]. Thus, it can be postulated that disturbance of cholesterol mechanism in either direction (decreased synthesis in the case of SLOS, or increased uptake by SCARB1 and conversion to androgen by SRD5A1 in our study) can have wide-ranging effects on neural development and function. The predicted increase in androgen levels due to increased expression of SRD5A1, on the other hand, is supported by our pilot study on the metabolites within the steroid hormone biosynthetic pathway which shows elevated testosterone in all 3 of the randomly selected autistic siblings relative to his respective nearly age-matched normal sibling as well as by other studies in the literature which show elevated androgen levels in the serum of autistic individuals, including females [14], [64], [71]. Our observation that at least 2 of the genes (SCARB1 and SRD5A1) that are involved in cholesterol import into the cell and testosterone metabolism exhibit increased expression in the autistic siblings offers a plausible explanation for elevated androgen levels in ASD.

The biological consequences of elevated testosterone on neurodevelopment and function are just beginning to be understood. While it has been known for more than 10 years that estrogens modulate synaptic plasticity in the hippocampus of female rats [78], it has only recently been shown that androgens likewise play a role in hippocampal synaptic plasticity, but in *both* males and females [79]. Furthermore, there is increasing evidence for the role of “neurosteroids” (which include DHEA and progesterone) in neurological functions, including rapid modulation of neurotransmitter receptors [80]. In contrast to testosterone, DHEA which has been shown to be lowered in ASD [81], plays a neuroprotective role countering the effect of stress-inducing steroids [82], [83]. Interestingly, we have observed that the plasma levels of DHEA were lower in several of the autistic siblings relative to their respective nonautistic siblings (unpublished data). Clearly, it will be important to further evaluate the levels of steroid hormones and related molecules in a broader sampling of individuals with ASD as well as to establish a correlation between these metabolite levels and aberrant expression of genes in this metabolic pathway.

Pathway analyses using Pathway Studio 5 also implicated involvement of female hormones in that the estrogens were among the small molecule regulators of the differentially expressed genes (**Fig. 3**). It is further noted that SRD5A1 is involved in sex determination [84]. Thus, the altered expression of genes involved in steroid hormone production and sexual dimorphism, coupled with the differential impact of male and female steroid hormones on brain development in male vs. female animals [78], [79] may, in part, underlie the approximately 4∶1 male to female ratio in ASD.

The schematic in **Figure 5** suggests that bile acid synthesis might also be affected by some of the differentially expressed genes in ASD, particularly SCARB1 and SRD5A1, which respectively internalize cholesterol and participate enzymatically in bile acid synthesis. This suggests that altered expression of genes in this pathway may also be responsible for the digestive and hepatic disorders associated with ASD. Indeed, in a separate case-control study of a large number of unrelated individuals (total of 116), hepatic cholestasis and fibrosis are strongly indicated on the basis of the gene expression profiles of the autistic probands vs. unrelated controls (Hu et al., Autism Research, 2009, in press). Changes in metabolite profiles thus may be predicted and tested on the basis of a functional analysis of altered gene interactions that arise from increases or decreases in gene expression within a specific metabolic pathway. Indeed, this would be a complementary approach to that used by James et al. who used targeted metabolite profiling of the methionine transmethylation and transsulfuration pathways to identify potential gene defects in ASD [22]. Such metabolomic analyses, guided by gene expression studies, may in turn lead to a diagnostic screen for ASD based on metabolite profiling of serum or other easily accessible tissues (e.g., steroid hormone, bile acid, or redox molecule assays).

Aside from genes involved in cholesterol metabolism and steroid hormone biosynthesis, we also confirmed the altered expression of several other novel genes that may play a role in the pathophysiology of autistic disorder (Fig. 2). The use of LCL cells as a surrogate tissue to study potential changes in brain gene expression that may mechanistically underlie autism or other neurological disorders is not unprecedented. Gene expression profiles of different brain regions have been shown to exhibit the highest similarity to whole blood [85]. Moreover, a meta-analysis of studies performed in blood and post-mortem brain demonstrated convergent gene expression changes [86], although further studies are warranted [87]. Because of their role in neuronal development, migration, and morphology (Table 2), we were particularly interested in confirming the differential expression of CXCR4, CD9, and GATA3. Although the chemokine receptor CXCR4 is most frequently associated with inflammatory processes and leukocyte trafficking in the immune system, recent studies show that it, along with the chemokine stromal cell-derived factor 1 (SDF-1), are important regulators of neuronal migration and axonal pathfinding, particularly in the cortex and cerebellum [88] where it is involved in the development and organization of Purkinje cells, which are notably deficient in ASD [4]. CD9 is yet another molecule involved in cell migration, both in the immune system as well as nervous system [89], [90], with its expression in Schwann cells regulated by axonal contact [91]. Interestingly, androgens have been shown to induce CD9 in human prostate [92], suggesting yet another mechanism for increased expression in autism. GATA3 is a transcription factor that is involved in both allergic inflammation (like CXCR4) and cytokine production [93], [94]. In addition, GATA3 has also been shown to be involved in the development of the central nervous system in mice [95] and the induction of dopamine beta-hydroxylase (DBH) in primary neural crest stem cells [96]. With respect to the latter activity, it is of interest to note that DBH genotype has been associated with autism in some families [97], [98] and that DBH activity has been noted to be elevated in a subgroup of autistic patients [99]. Like the 3 genes discussed above, NFKBIZ, a nuclear regulator of NFKB activity, is also involved in inflammation and immunity. In particular, it is induced upon stimulation of the innate immune system [100] and, in turn, stimulates IL-6 production [101]. These characteristics of NFKBIZ are noteworthy in light of studies by Pardo and colleagues demonstrating activation of the innate immune system in brain tissues from autistic patients, with a notable increase in IL-6 [12]. Thus, the elevation of NFKBIZ in peripheral cells derived from autistic probands may be a reflection of a systems-wide activation of the innate immune system in autism, providing strong support for the use of LCL as a surrogate model to examine gene dysregulation in ASD.

In summary, gene expression profiling of LCL from autistic and nonautistic siblings reveals alteration of genes involved in both metabolic and signaling pathways in ASD that is consistent with the known pathophysiology of autism which includes inflammation as well as disturbances in axon guidance, neuronal survival, and differentiation, biological themes also implicated in our earlier study on monozygotic twins discordant in diagnosis and severity of autism [43]. The involvement of genes affecting both the immune and nervous systems, coupled with the pleiotropic effects of dysregulated steroid hormone biosynthesis, may further explain some of the systemic disorders associated with autism. The genes, metabolites, and pathways identified in this study moreover suggest novel targets for therapeutics. Thus, gene expression profiling, which provides a global view of functional gene networks in the context of living cells from individuals with ASD, not only allows for the elucidation of compromised pathways but also provides a meaningful and complementary (with respect to genetics) approach towards understanding the complex biology of ASD.

## Methods

### Cell Culture

Lymphoblastoid cell lines (LCL) derived from lymphocytes of autistic and normal siblings were obtained from the Autism Genetic Resource Exchange (AGRE) and cultured in RPMI 1640 with 15% fetal bovine serum and antibiotics in a humidified incubator under 5% CO_2_. **Supplementary Table 1** summarizes the demographic profile of subjects whose LCL were analyzed in this study.

### Selection of samples

**Figure 6:**
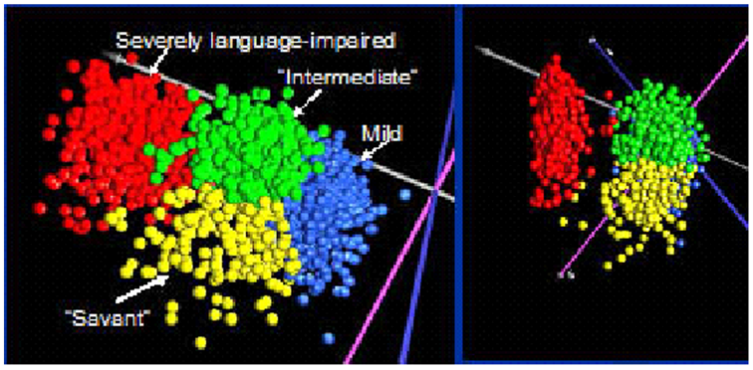
Separation of 1351 autistic probands (each represented by a point) into phenotypic groups on the basis of principal components analysis (PCA) of 123 scored items on the Autism Diagnostic Interview-Revised (ADIR) questionnaires for each individual which was obtained from the AGRE phenotypic database. PCA divided the autistic individuals into 2 main groups. Hierarchical clustering of the ADIR data (data not shown) revealed that individuals in the smaller group were characterized by higher severity scores on spoken language items on the ADIR. These individuals are represented by the red points in the PCA. Hierarchical clustering also suggested 3 other phenotypic groups that were characterized by lower severity scores across all items (individuals coded blue), higher frequency of savant skills (individuals coded yellow), and intermediate severity across all items (individuals coded green). To restrict sample heterogeneity, this study used LCL only from individuals with severe language impairment (coded red) as identified by these 2 cluster analyses. Detailed methods used for the identification of distinct ASD behavioral phenotypes based on cluster analyses of ADIR scores are described by Hu and Steinberg (Autism Research (2009) in press).

To reduce the heterogeneity of the samples for gene expression analyses, we used a novel clustering procedure to identify phenotypically distinct groups of individuals on the basis of severity associated with 123 items on the Autism Diagnostic Interview-Revised scoresheets. This procedure, described in another manuscript (submitted for publication), resulted in separation of the autistic individuals into 4 phenotypic subgroups, as illustrated in **Fig. 6**. For this study, we selected autistic male individuals from the distinct subgroup associated with severe language impairment, each of whom had a male sibling who was not affected by autism who served as a control in a paired statistical analysis of gene expression data derived from LCL of the respective siblings. To further reduce the heterogeneity within the samples and eliminate confounding factors due to co-existing conditions or known genetic abnormalities, LCL from females, individuals with specific genetic and chromosomal abnormalities (e.g., Fragile X, chromosome 15q11-q13 duplication) and with diagnosed co-morbid disorders (e.g., bipolar disorder, obsessive compulsive disorder), and those born prematurely (<35 weeks of gestation) were excluded from this study.

### DNA Microarray Analysis and Data Sharing

RNA was isolated from LCL 3 days after the last passage using TRIzol Reagent (Invitrogen) according to the manufacturer's protocol. Fluorescently labeled sample cDNA was obtained by incorporation of amino-allyl dUTP during first-strand synthesis, followed by coupling to the ester of Cyanine (Cy)-3 as previously described [43]. Stratagene Universal human reference RNA was used as a common reference RNA sample for all hybridizations, in which the reference cDNA was labeled with Cy-5 dye. For two-color DNA microarray analyses, sample and reference cDNA were co-hybridized onto a custom printed microarray containing 39,936 human PCR amplicon probes derived from cDNA clones purchased from Research Genetics (Invitrogen ). After hybridization and washing according to published procedures [43], the microarrays were scanned for fluorescence signals using a Genepix 4000B laser scanner. Normalized gene expression levels were derived from the resulting image files using TIGR SpotFinder, MIDAS, and MeV analysis programs which are all part of the TM4 Microarray Analysis Software Package available at www.tm4.org. Within MeV, the Significance Analysis of Microarray (SAM) module [102] was employed to obtain statistically significant differentially expressed genes using a one-class SAM analysis of the log2 ratios of relative expression data from the autistic and nonautistic sib pairs. Details of this analysis as well as the raw and normalized intensity data for all samples have been deposited into GEO (Accession # GSE15451).

### Quantitative PCR Analysis

Select genes were confirmed by real time RT-PCR on an ABI Prism 7300 Sequence Detection System using Invitrogen's Platinum SYBR Green qPCR SuperMix-UDG with ROX. These included genes involved in cholesterol and steroid hormone metabolism as well as genes implicated in nervous system development that were enriched within the top biological functions identified by Ingenuity Pathway Analysis (Table 2). Total RNA (same preparations used in microarray analyses) was reverse transcribed into cDNA using the iScript cDNA Synthesis Kit (Bio-Rad, Hercules, CA). Briefly, 1 µg of total RNA was added to a 20 µl reaction mix containing reaction buffer, magnesium chloride, dNTPs, an optimized blend of random primers and oligo(dT), an RNase inhibitor and a MMLV RNase H+ reverse transcriptase. The reaction was incubated at 25°C for 5 minutes followed by 42°C for 30 minutes and ending with 85°C for 5 minutes. The cDNA reactions were then diluted to a volume of 50 µl with water and used as a template for quantitative PCR.

PCR primers for genes identified by microarray analysis as differentially expressed were selected for specificity by the National Center for Biotechnology Information Basic Local Alignment Search Tool (NCBI BLAST) of the human genome, and amplicon specificity was verified by first-derivative melting curve analysis with the use of software provided by PerkinElmer (Emeryville, CA) and Applied Biosystems. Sequences of primers used for the real-time RT-PCR are given in the **Supplemental Table S2**.

Quantitative reverse transcriptase-PCR analyses (qRT-PCR) were performed on a representative set of 5 pairs of case-controls from the sib pair analyses, with quantification and normalization of relative gene expression using universal 18S rRNA primers, with samples normalized to their 18S rRNA standard curves. The qPCR reactions were done in triplicate. A one-sample t-test was used to determine significance of differential expression across the 5 paired samples.

### Pathway and Functional Analyses

The datasets of differentially expressed genes between autistic probands and unaffected siblings were analyzed using Ingenuity Pathway Analysis and Pathway Studio 5 to identify molecular and cellular processes, high level functions, disorders, and small molecules associated with the gene regulatory networks. DAVID Bioinformatics Resources (http://david.abcc.ncifcrf.gov) was also used for additional functional annotation [65]. In both types of analyses, statistically significant functions were determined using the Fisher Exact test.

### Metabolic profiling of steroid hormones in LCL

Metabolites were extracted from LCL using acetonitrile and analyzed by isotope dilution liquid chromatography-photospray ionization tandem mass spectrometry, a highly sensitive method which has been developed for the simultaneous determination of 11 steroids [103]. Briefly, 300 µl of acetonitrile containing the deuterated internal standards is added to the cell pellet containing 2×10^8^ cells, vortexed, and incubated for 30 min at RT. Two hundred µl of water is then added along with internal standards and the mixture is centrifuged to precipitate the proteins. After protein removal, 350 µl of supernatant is diluted with 1.4 ml of water and 1.5 ml of the resulting solution is injected into the LC-APPI-MS/MS (Applied Biosystems API-5000 triple quadrupole mass spectrometer equipped with an atmospheric pressure photoionization source).

## Supporting Information

Table S1 Demographic profile of subjects
(0.02 MB XLS)

Table S2 Primer sequences used for qRT-PCR
(0.03 MB DOC)
