# Supplementary material for: Gene Expression Profiling of Lymphoblasts from Autistic and Nonaffected Sib Pairs: Altered Pathways in Neuronal Development and Steroid Biosynthesis
Source: PLoS One. 2009 Jun 3;4(6):e5775. doi: 10.1371/journal.pone.0005775 (PMC2685981; doi:10.1371/journal.pone.0005775)
Supplement: Table S2 — Primer sequences used for qRT-PCR (0.03 MB DOC) [file pone.0005775.s002.doc]

Supplemental Table 2. Sequences of primers used for qRT-PCR analyses

| **Gene Symbol** | **Forward Primer (5' --> 3')** | **Reverse Primer (5' --> 3')** |
| --- | --- | --- |
| 18S | CCGCAGCTAGGAATAATGGA | CCCTCTTAATCATGGCCTCA |
| CD9 | GGGCGTGGAACAGTTTATCTC | GTCGAAGACCTCTTTGATGG |
| CXCR4 | TTCCTGCCCACCATCTACTC | CTTGTCCGTCATGCTTCTCA |
| GATA3 | CACAACCACACTCTGGAGGA | TTTTTCGGTTTCTGGTCTGG |
| NFKBIZ | ACTCGGAACTTGGAGAACGA | GGAGCTCTCTGCTGAATGGA |
| SCARB1 | AGGCCATTCAGGCCTATTCT | CTGGCTCACGGTGTCCTC |
| SRD5A1 | GGCTTTTGCTTTCTTCACGTT | ATAGAGAAGCGCCATTGGAA |
